# High expression of TREM2 promotes EMT via the PI3K/AKT pathway in gastric cancer: bioinformatics analysis and experimental verification

**DOI:** 10.7150/jca.55077

**Published:** 2021-04-02

**Authors:** Chunmei Li, Xiaoming Hou, Shuqiao Yuan, Yigan Zhang, Wenzhen Yuan, Xiaoguang Liu, Juan Li, Yuping Wang, Quanlin Guan, Yongning Zhou

**Affiliations:** 1 Department of Gastroenterology, The First Hospital of Lanzhou University, Lanzhou, China.; 2 Key Laboratory for Gastrointestinal Diseases of Gansu Province, The First Hospital of Lanzhou University, Lanzhou, China.; 3 Department of Oncology, The First Hospital of Lanzhou University, Lanzhou, China.; 4 Department of medical laboratory, The First Hospital of Lanzhou University, Lanzhou, China.; 5 The First School of Clinical Medicine, Lanzhou University, Lanzhou, China.; 6 Department of Oncology Surgery, The First Hospital of Lanzhou University, Lanzhou, China.; 7 Department of Rheumatology, The First Hospital of Lanzhou University, Lanzhou, China.; 8 Department of Gastroenterology, Gansu Provincial Hospital, Lanzhou, China.

**Keywords:** TREM2, gastric cancer (GC), EMT, PI3K/AKT pathway, bioinformatics

## Abstract

**Background:** To date, the pathogenesis of gastric cancer (GC) remains unclear. We combined public database resources and bioinformatics analysis methods, explored some novel genes and verified the experiments to further understand the pathogenesis of GC and to provide a promising target for anti-tumor therapy.

**Methods:** We downloaded the chip data related to GC from the Gene Expression Omnibus (GEO) database, extracted differentially expressed genes (DEGs), and then determined the key genes in the development of GC via PPI networks and model analysis. Functional annotation via GO and KEGG enrichment of DEGs was used to understand the latent roles of DEGs. The expression of the triggering receptor expressed on myeloid cells 2 (TREM2) gene in GC cell lines was verified via RT-PCR and western blotting. Moreover, the CCK-8, wound healing assay, and transwell migration and invasion assays were used to understand the changes in the proliferation, migration, and invasion abilities of GC cells after silencing TREM2. Western blotting verified the interaction between TREM2 and PI3K predict of the string website, as well as the effect of TREM2 on EMT. Finally, a lung metastasis model was used to explore the relationship between TREM2 and metastasis.

**Results:** Our study identified 16 key genes, namely BGN, COL1A1, COL4A1, COL5A2, NOX4, SPARC, HEYL, SPP1, TIMP1, CTHRC1, TREM2, SFRP4, FBXO32, GPX3, KIF4A, and MMP9 genes associated with GC. The EMT-related pathway was the most significantly altered pathway. TREM2 expression was higher in GC cell lines and was remarkably associated with tumor invasion depth, TNM stage, histological grade, histological type, anatomic subdivision, and *Helicobacter pylori* state. Knockdown of TREM2 expression inhibited the proliferation, migration, and invasion of GC cells as well as the progression of EMT by PI3K/AKT signaling *in vitro*. In addition, lung metastasis were decreased *in vivo*.

**Conclusions:** We identified some important genes associated with the progression of GC via public database analysis, explored and verified the effects of proto-oncogene TREM2 on EMT via the PI3K/AKT pathway. TREM2 may be a novel target in the GC therapy.

## Introduction

Gastric cancer (GC) is a fatal disease that seriously affects people's health. In 2018, 730,000 people died of GC worldwide 1. Most patients with GC are diagnosed at an advanced stage and lose the opportunity for surgery. Limited therapy options are available, such as chemotherapy, radiotherapy, and targeted therapy, but outcomes are not satisfactory because of the heterogeneity and diversity of GC 2. Therefore the 5-year survival rate of patients with GC is low. We should actively search for important molecules associated with the carcinogenesis of GC, deeply understand the pathogenesis of GC, and provide more novel and effective targets for the diagnosis and treatment of GC.

In the past decade, the development of microarray technology and bioinformatics analyses tools has facilitated the discovery of some novel genes and pathways in the development of tumor from multiple levels as well as exploration of biomarkers related to tumor survival, prognosis, and diagnosis 3-7. Many newly discovered genes have also been confirmed in cell and animal experiments 8,9. This new idea provides a new direction for the study of the detailed mechanism of tumorigenesis. We used the data of patients with GC in the public database Gene Expression Omnibus (GEO) to explore the functional aggregation of differentially expressed genes (DEGs) and to search for significantly altered metabolic pathways. We found key genes in the progression of GC by constructing protein-protein interaction (PPI) networks and modular analysis and further explored and validated the genes that may be therapeutic targets.

Bioinformatics analysis has revealed that the protein-coding gene triggering receptor expressed on myeloid cells 2 (TREM2) was a key gene in GC progression. Previous studies have reported that TREM2 has roles in the degree of the inflammatory response under infection 10,11, osteoclast differentiation during bone reconstruction 12, and the occurrence of Alzheimer's disease 13,14. With the rapid emergence of different studies, TREM2 has been reported to affect the pathogenesis of some types of tumor 15-20. For example, Kim et al. proved that TREM2 suppresses tumor development *in vivo* and *in vitro* assays by targeting Wnt1/β-catenin and Erk signaling and serves as a therapeutic target for colorectal cancer 17. TREM2 affects carcinogenesis by PI3K/AKT/β-catenin signaling in hepatocellular carcinoma 20. Besides, silencing TREM2 suppresses cell proliferation, invasion, and migration and significantly increases the apoptosis of glioma cell lines, suggesting that TREM2 may be a novel therapeutic target 19. Nonetheless, the role of TREM2 in GC remains unclear.

In our study, we analyzed the public database resources using bioinformatics methods to find the key genes that may participate in the occurrence and development of GC and reviewed the connection between these key genes and GC. Furthermore, we determined that the key gene TREM2 is related to a variety of clinicopathological features and overall survival (OS) in patients with GC. We explored and verified that TERM2 can promote the EMT process via the PI3K/AKT pathway at the bioinformatic and experiment levels.

## Materials and methods

### Data processing and DEGs identification

Two gene expression microarray datasets, namely GSE72305 and GSE103236, were acquired from the GEO database. The GSE72305 dataset was from the Agilent GPL15314 platform and included two normal gastric tissues, five cases of primary GC specimens without lymph node metastasis (LNM), and five cases of primary GC specimens with LNM. The GSE103236 dataset was from the Agilent GPL4133 platform and included ten pairs GC and normal adjacent tissues. The LIMMA package in R language, a linear regression model, was used to explore the DEGs in GC tissues and adjacent tissues 21. A *p* value of <0.05 and logFC (fold change) of >1.0 were set as thresholds to screen DEGs. The intersection genes were graphically overlapped using online Venn diagram tools (http://bioinformatics.psb.ugent.be/ webtools/Venn/).

### Functional enrichment analysis

The enrichment analyses of the DEGs, consisting of Gene Ontology (GO) function and Kyoto Encyclopedia of Genes and Genomes (KEGG) pathway, were implemented using the FunRich software. These analyses provide gene functional classification and comprehensive functional explanation for researchers to comprehend biological characteristics 22.

### PPI network construction, module analysis, and hub gene selection

Based on STRING (http://string-db.org. Version 10.0) online tool 23, the PPIs of the DEGs were screened with a confidence score of ≥0.7. Then, the PPI network was visualized using the Cytoscape software (version 3.5.1). PPI networks were drawn using Cytoscape, and the most significant module in the PPI networks was identified using MCODE 24. The criteria for selection were as follows: MCODE scores >5, degree cut-off = 2, node score cut-off = 0.2, Max depth = 100, and k-score = 2. Finally, the node of high degree of >10 was selected as the hub genes.

### Survival analysis of key genes

The Kaplan-Meier plotter online database (http://kmplot.com/analysis/) was used to calculate the OS of individual key gene, which classified the GC patients into high-expression and low-expression groups based on the expression median of the key gene.

### Patients sample from TCGA database

We downloaded clinicopathological data including patients' sex, age, histologic type, histologic grade, TNM stage, and anatomic subdivision from TCGA database, 393 cases about patient with gastric adenocarcinoma, 175 cases about patient with gastric adenocarcinoma and *HP* infection status. The correlation between TREM2 and clinicopathological features were analyzed by these database, separately.

### Cell culture

Normal gastric epithelium cell line (GES-1) and human GC cell lines (SGC-7901, BGC-823, MGC-803, HGC-27, and MKN-45) were purchased from Chinese Academy of Sciences (Shanghai, China). Cells were cultured in RPMI 1640 medium supplemented with 10% fetal bovine serum (FBS), 100 mg/mL streptomycin, and 100 U/mL penicillin. Cells were maintained in a humidified incubator with 5% CO_2_ at 37 °C and logarithmic growth.

### Lentivirus transduction

The small hairpin RNA (shRNA) oligonucleotides against human TREM2 (target region 1, 5′-GAGCCTCTTGGAAGGAGAAAT-3′; target region 2, 5′-CACAGCCATCACAGACGATAC -3′) and control shRNA non-specific control oligonucleotides were annealed and inserted into the BamH I-EcoR I site of pPLK/GFP+puro vector. Viral packaging was implemented in HEK293T cells using the GM easyTM Lentiviral Packaging Kit (Genomeditech, Shanghai). The supernatant conditioned viral particles were harvested at 48 and 72 h after transfection and the filtered through 0.45 μm filters. After viral infection, GC cells were incubated with filtered conditioned media for 8 h. Puromycin was then added to select lentivirus-infected cells. After 2 weeks, experiments were performed.

### Cell proliferation assay

Cell proliferation was analyzed using the Cell Counting Kit-8 (CCK-8) assay. Different groups of GC cells (1 × 10^3^) were seeded into 96-well plates and were cultured for 24, 48, 72, 96, and 120 h. Then, cells were incubated with 10 μL of CCK-8 solution for 2 h. The absorbance in each well was measured at OD450 using a microplate reader RT-6000 (Rayto, China).

### Wound healing assay

In brief, the transfected cells were seeded into 6-well plates at a concentration of 3-7 × 10^5^ cells/ml. When cells were cultured to approximately 90% confluency, a 200 μL pipette tip was used to make a straight wound on the confluent monolayer. The cells were then culturing on serum-free medium for 48 h. The wounded monolayer was washed with phosphate-buffered saline (PBS) and photographed using an inverted microscope.

### Transwell migration and invasion assays

Migration and invasion assays were performed in 24-well plates with inserts (8 μmol/L pore size, Corning) without or with Matrigel. Cancer cells (2 × 10^4^ cells/well) were added into the upper chambers in serum-free media. Meanwhile, RPMI 1640 containing 5% FBS was added to the lower chambers. After incubation at 37 °C in 5% CO_2_ for 24 h (migration assay) or 36 h (invasion assay), the upper chamber was cleaned with a cotton swab and the lower chamber was fixed with 4% paraformaldehyde, dyed with 0.1% crystal violet, and then washed with water for three times. An inversion microscope (Olympus CKX53, Japan) was used to image the cells under a microscope. The number of cells per field was calculated using the Image J Version 1.48 software (National Institutes of Health, Bethesda, MD).

### Real-time polymerase chain reaction (RT-PCR)

RNA was extracted from GC cells using the Trizol Kit (Invitrogen, CA) according to a standard protocol. Biophotometer B-500 (Metash, Shanghai) was used to determine the quality and quantity of the isolated RNA. Subsequently, complementary DNA was synthesized using the PrimeScript™ RT reagent Kit with gDNA Eraser (Takara). The expression level of TREM2 was detected using RT-PCR with TB Green™ Premix Ex Taq II (Takara) according to the manufacturer's instructions. GAPDH was used as a reference for normalization. The primer sequences were as follows: TREM2 primer: F, 5′-ACTACTCTGCCTGAACAC-3′ and R, 5′-GCTAAATATGACAGTCTTGGA-3′, GAPDH primer: F, 5′-GTCTCCTCTGACTTCAACAGCG-3′ and R, 5′-ACCACCCTGTTGCTGTAGCCAA-3′. The fold change of TREM2 was calculated by the 2^-ΔΔCT^ method.

### Western blot analysis

Total protein was extracted using the RIPA Lysis Buffer and PMSF, phosphatase inhibitor (Sangon Biotech, Shanghai) following the manufacturers' instructions, followed by centrifugation for 15 min at 12,000 rpm. The bicinchoninic acid assay was used to measure total protein concentrations. Proteins (30 µg per lane) were separated on SDS-PAGE gels and transferred on to PVDF membranes. Membranes were blocked with 5% non-fat milk and incubated with primary antibodies at 4 °C overnight. Then, cells were incubated with an HRP-conjugated secondary antibody. The bands were detected with enhanced chemiluminescence and visualized using a Fluorescent and Chemiluminescence Gel Imaging System (Peiqing Science and Technology Co., Ltd.). The primary antibodies were purchased from ABcam (Cambridge, UK). Antibody dilutions were 1:800 for the anti-TREM2 antibody; 1:1000 for the anti-E-cadherin antibody; 1:800 for the anti-N-cadherin antibody; 1:1000 for the anti-Vimentin antibody; 1:500 for the anti-PI3K antibody; 1:500 for the anti-AKT antibody; 1:500 anti-p-PI3K antibody; and 1:500 for the anti-p-AKT antibody. The Image J Version 1.48 software was used to quantify protein expression, and GAPDH was used as a loading control.

### Immunofluorescence

Cells were fixed with 4% paraformaldehyde for 20 min and washed with PBS three times for 3 min. Cells were then permeabilized at room temperature with 0.5% Triton X-100 for 20 min and washed with PBS. Goat serum was used for blocking at room temperature for 30 min. Cells were incubated with 1:50 dilution of the anti-E-cadherin primary antibody (ABclonal , A3044) overnight at 4 °C and then washed with PBS. The fluorescent secondary antibody [1:200, goat anti-Rabbit Alexa 594 (ZSGB‐BIO)] was added and the cells were incubated at room temperature in the dark for 2 h. Cells nuclei were visualized via staining with DAPI (Sigma Aldrich) in the dark for 2 min and were observed under an inverted fluorescent microscope.

### Pulmonary metastasis model

Ten BALB/C nude mice were purchased from Hunan SJA Laboratory Animal Co., Ltd. Five-week-old male nude mice were divided into two groups, and 1 × 10^6^/100 µL of BGC-823 shCtrl or BGC-823 shTREM2 #2 cells were injected via the tail vein. After 1 month, all mice were sacrificed. The tumors in lung tissues were observed under a microscope. Experiments conformed to the local ethics committee and the Use Committee for Animal Care and were performed in compliance with institutional guidelines.

### Statistical analysis

All date analysis was performed using the Statistical Package for the Social Sciences (SPSS) 23.0 software and the GraphPad Prism v. 7.01, software package. The results are expressed as means ±standard deviation (SD) unless otherwise mentioned. Correlations between TREM2 expression and clinicopathological factors were analyzed using the Chi-squared test. The statistical comparisons were evaluated using the Student's t-test and one- or two-way ANOVA. A *P*< 0.05 was considered statistically significant. All experiments were repeated three times.

## Results

### Identification of DEGs in GC

**Figure 1:**
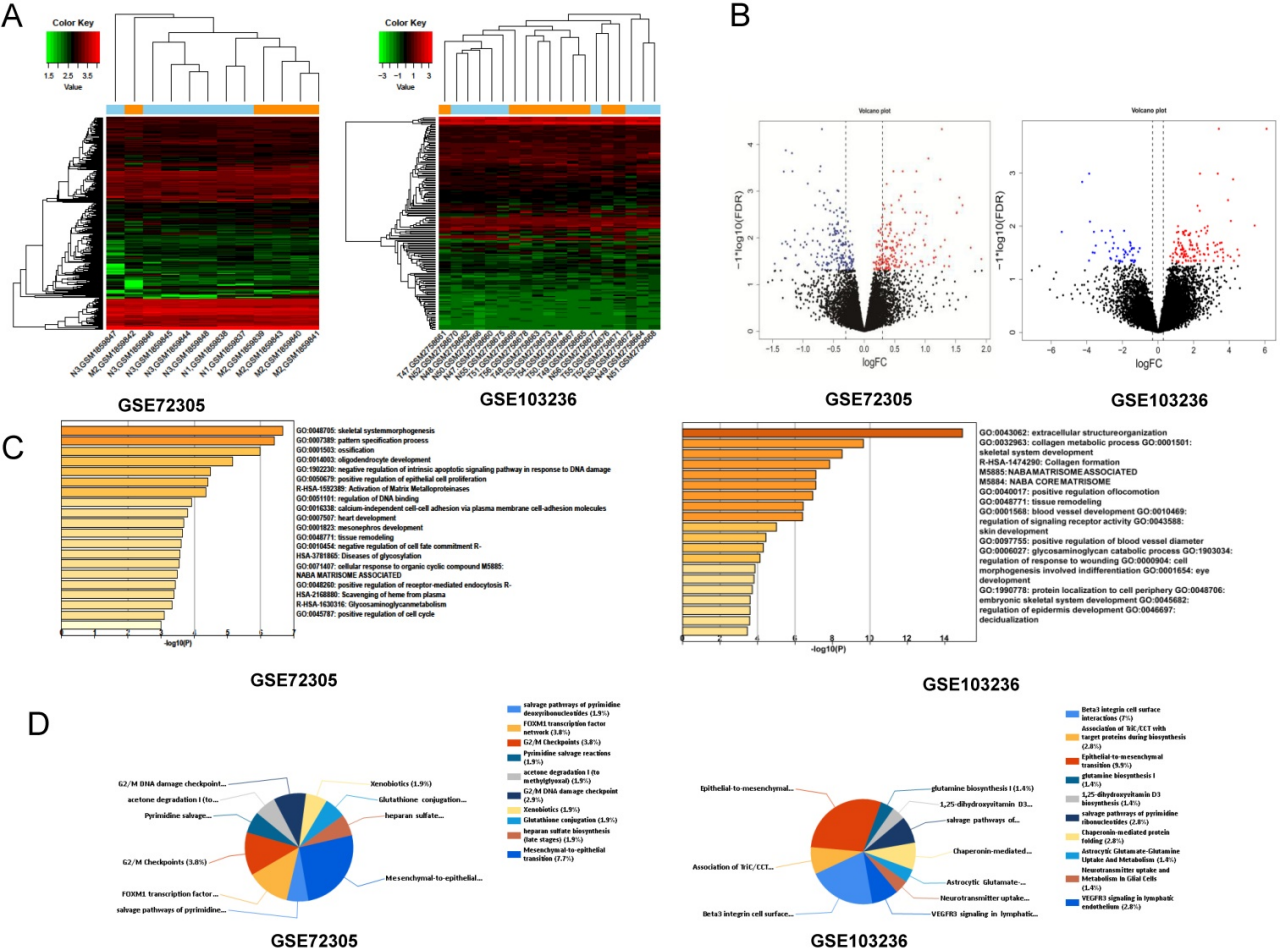
Identification of key genes and function and pathway analysis about GSE72305 and GSE103236. (A) The expression profile of gastric cancer related expressed genes. (B) Volcanic plot of gastric cancer-related differentially expressed genes (DEGs) respectively. (C, D) The biological process of EDGs by Gene Ontology (GO) and the biological pathways analysis of DEGs by Kyto Encyclopedia of Gene and Genomes (KEGG) enrichment about GSE72305 and GSE103236.

After standardization of the chips, we identified 146 and 127 upregulated DEGs and 150 and 48 downregulated DEGs in the GSE72305 and GSE103236 datasets (Figure 1A, 1B), respectively, by the LIMMA package.

### Functional enrichment analysis of DEGs

We analyzed biological classification and functional enrichment using the FunRich software. The biological functions of DEGs from GSE72305 dataset were mainly involved in skeletal system morphogenesis and ossification, oligodendrocyte development, negative regulation of intrinsic apoptotic signaling pathway in response to DNA damage, positive regulation of epithelial cell proliferation (Figure 1C). Meanwhile, the biological pathways were mainly associated with the EMT (7.7%), G2/M checkpoint (3.8%), FOXM1 transcription factor network (3.8%), G2/M DNA damage checkpoint (2.9%) (Figure 1D). Moreover, the biological process of DEGs from GSE103236 were mainly involved in extracellular structure organization, collagen metabolic process, skeletal system development, and collagen formation (Figure 1C), and the biological pathway mainly associated with EMT (9.9%), beta-3 integrin cell surface interactions (7%), VEGFR3 signaling in lymphatic endothelium (2.8%), and chaperonin-mediated protein folding (2.8%) (Figure 1D). Among these singling pathways of the two chips, it was observed that the EMT singling pathway was important in DEG-related pathways.

### Screening of the key gene

**Figure 2:**
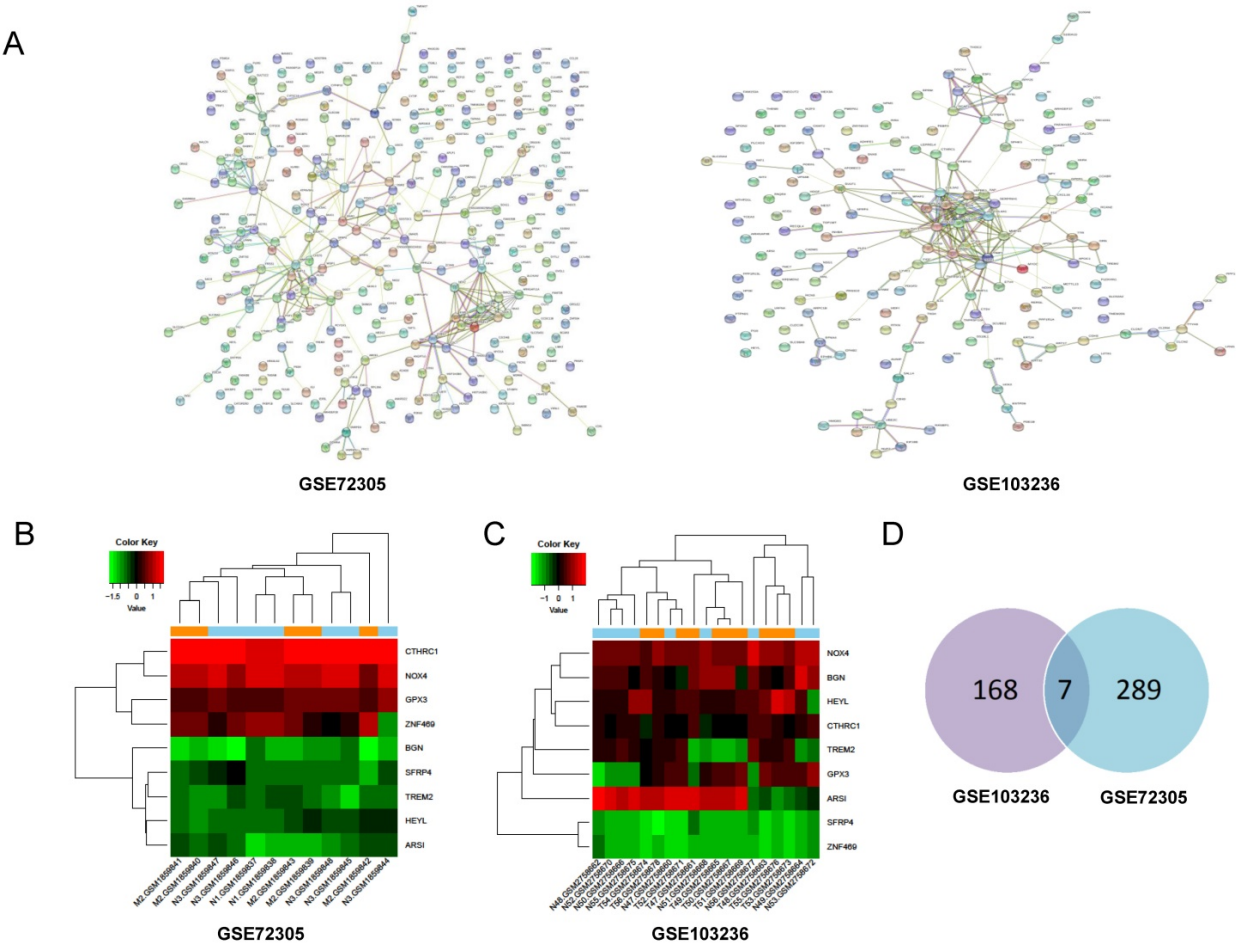
Screening of hub genes by PPI network and model analysis. (A) Protein-protein interaction network of differentially expressed genes in GSE72305 and GSE103236. The different colored lines indicate the different evidences demonstrating the interaction. (B, C) The heatmap about expression profile of nine common genes in GSE72305 and GSE103236. (D) Seven common DEGs in GSE72305 and GSE103236 by Venn diagrm software.

**Figure 3:**
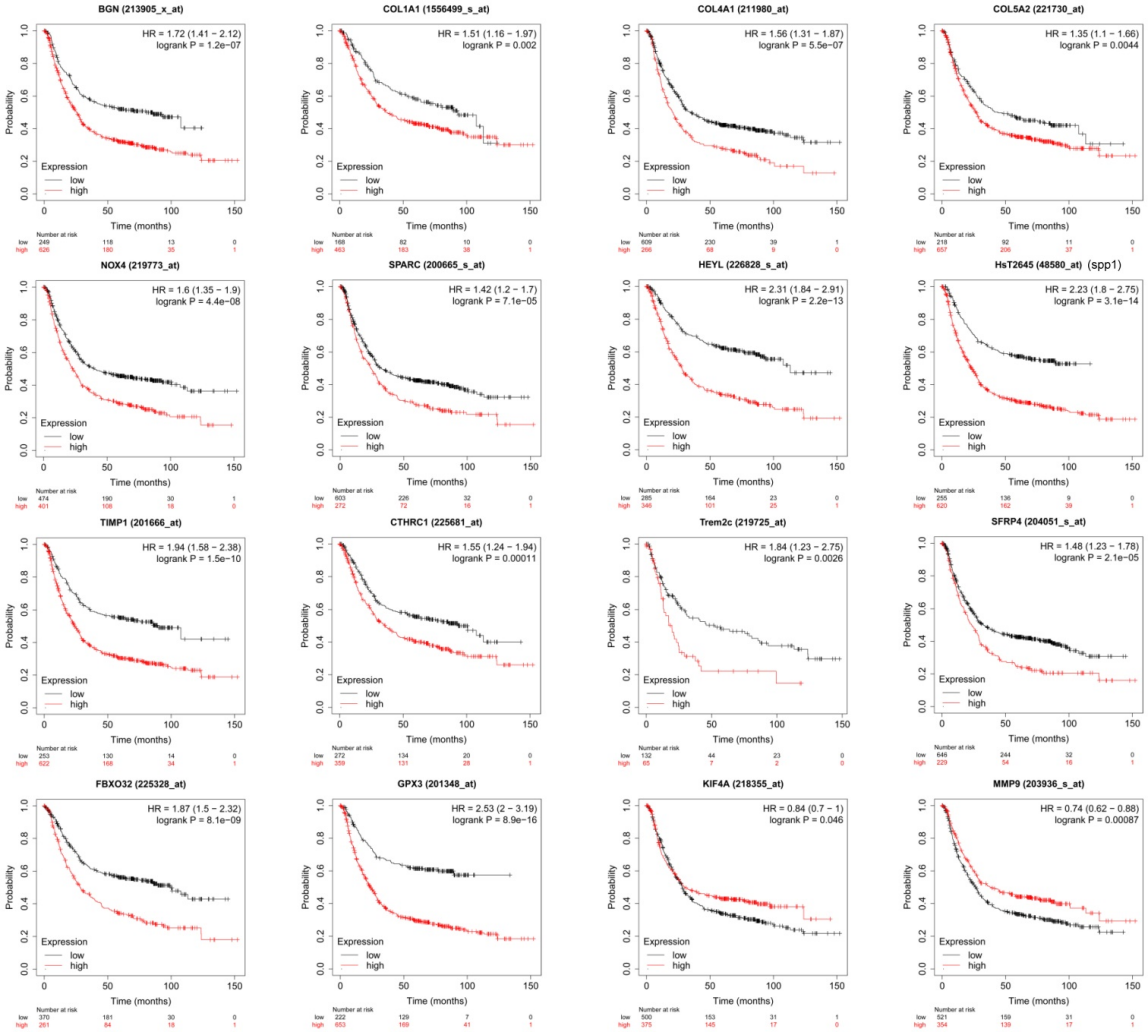
The survival analysis of the key genes in GC patients. The online Kaplan-Meier plotter tool was used to analysis the prognostic information of the 16 key genes. p<0.05 was considered statistically significant.

Through PPI network construction and module analysis of DEGs (Figure 2A), we found 10 hub genes from two chips including BGN, COL4A1, COL5A2, SPP1, TIMP1, KIF4A, MMP9, FBXO32, COL1A1, and SPARC. Meanwhile, we found 9 common genes (Figure 2B, 2C) and 7 common DEGs in GSE72305 and GSE103236 (Figure 2D). To avoid missing important molecules, we analyzed 7 common DEGs shared by the two chips together with hub genes namely TREM2, CTHRC1, BGN, NOX4, GPX3, HEYL, and SFRP4. Therefore, these 16 genes were included in the next-step analysis. Furthermore, in addition to GPX3 other genes were highly expression in GC tissue compared with normal gastric mucosal tissue with the cancer genome atlas (TCGA) database as the validation dataset (Additional file: Figure S1). Besides, these genes expression were closely associated with GC occurrence, development (Table 1) and prognosis by the Kaplan-Meier plotter online database (Figure 3). Most genes have been verified to take part in the occurrence and development of GC. Inflammation related gene TREM2 related to clinicopathological characteristics and prognosis in GC, but specific mechanism is not clear. We will further explore it by experiments.

### TREM2 overexpression in GC

**Figure 4:**
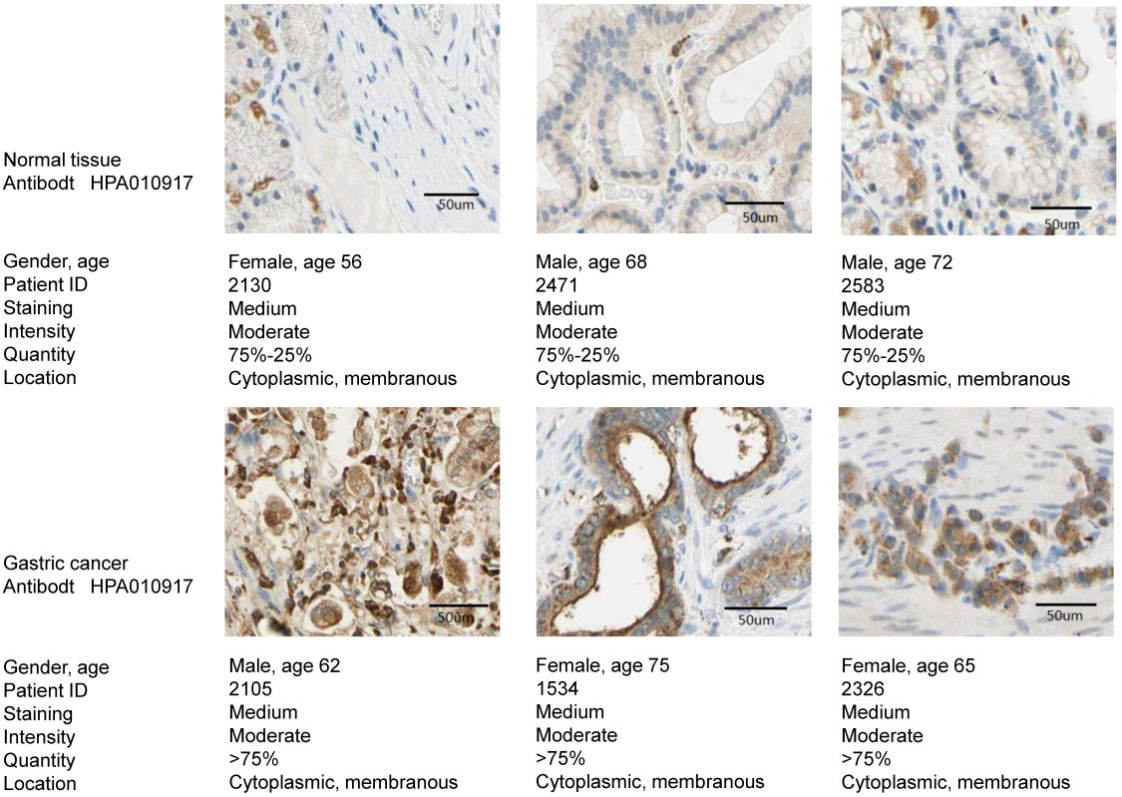
The TREM2 expression level between GC sample and normal tissue with immunohistochemistry analysis from the human protein ATLAS.

**Figure 5:**
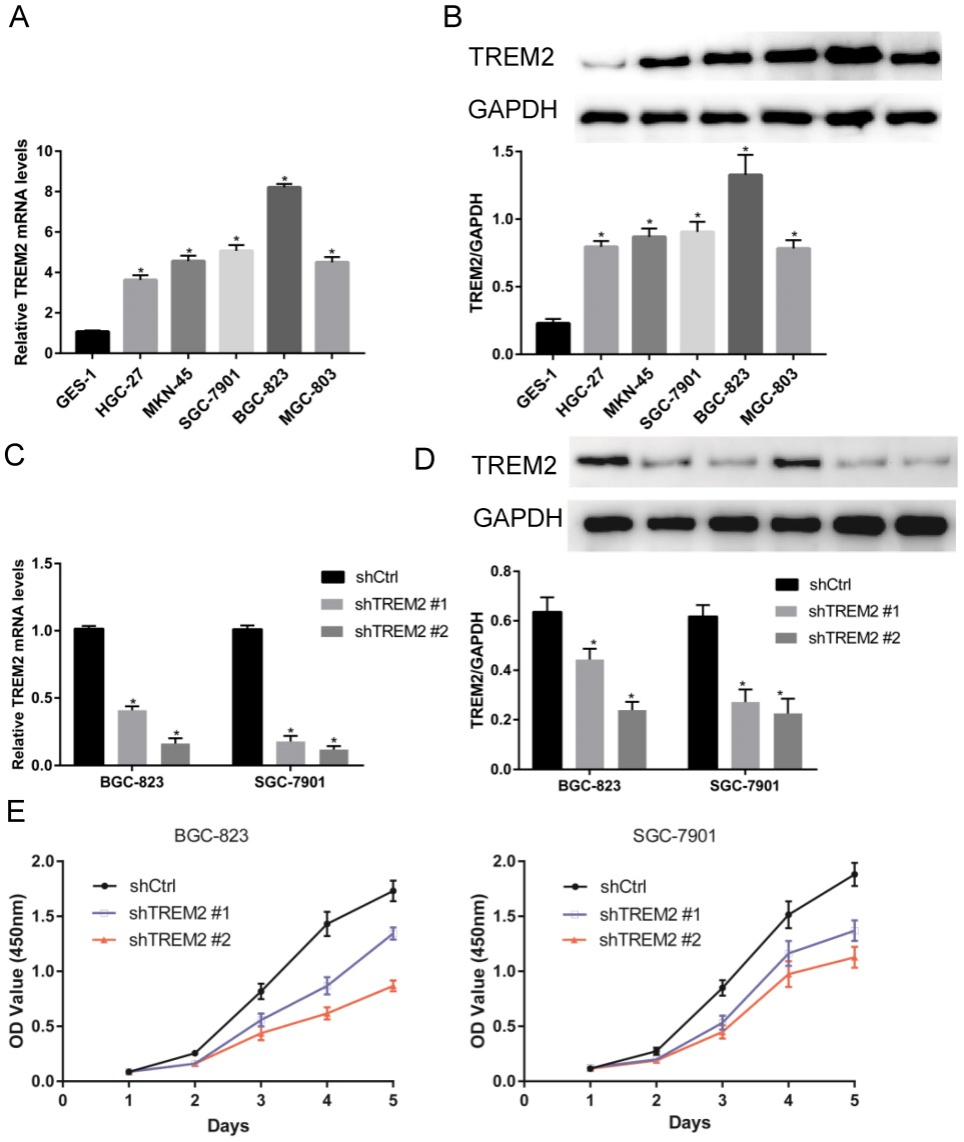
The expression of TREM2 in GC and affected cell proliferation. (A, B) TREM2 is high expression in GC cell lines at mRNA and protein levels. (C, D) Verified the ability of silence, shTREM2# 2 is more effectivity than shTREM2# 1 both in BGC-823 and SGC-7901 by RT-PCR and Western Blot. (E) The proliferation of BGC-823 and SGC-7901 were inhibited after knockdown of TREM2 measured by CCK8.

The expression of TREM2 was remarkably elevated in GC tissues than in paracancerous normal tissues in both GEO and TCGA databases. From The human protein ATLAS, the TREM2 expression level in GC sample were improved than normal tissue with immunohistochemistry analysis (Figure 4). To further confirm the expression of TREM2, we used RT-PCR and western blotting to verify the expression of TREM2 in different GC cell lines. The mRNA and protein expression levels of TREM2 were significantly improved in the GC cell lines SGC-7901, BGC-823, MGC-803, MKN-45, and HGC-27 compared with in GES-1 (Figure 5A, 5B). These results demonstrated that TREM2 is overexpressed in GC.

### Correlation between TREM2 and clinicopathological features

393 cases about patient with gastric adenocarcinoma, 175 cases about patient with gastric adenocarcinoma and *HP* infection status, were used to analyze. The expression of TREM2 was associated with the depth of tumor invasion (*P* < 0.05), TNM stage (*P* < 0.05), histologic grade (*P* < 0.0001), histologic type (*P* < 0.05), and anatomic subdivision (*P* < 0.01) in 393 patients (Table 2). In the analysis of 175 patients with* HP* infection status, TREM2 was correlated with tumor invasion depth (*P* < 0.05), histologic grade (*P* < 0.0001), *HP* infection status (*P* < 0.05). This difference may be related to the different sample size in the data set. *HP* infected patients were detected in the TREM2 low group with 25%, while 75% in TREM2 high group, *HP* negative patients were detected in the TREM2 low group with 51.61%, while 48.39% in TREM2 high group (*P* < 0.05) (Additional file: Table S1). There are not associated between TREM2 expression and age, gender, or LNM, distant metastasis. Above results indicate that the expression of TREM2 is related to clinicopathological features of patients with GC.

### TREM2 knockdown inhibits GC cell proliferation, migration and invasion *in vitro*

**Figure 6:**
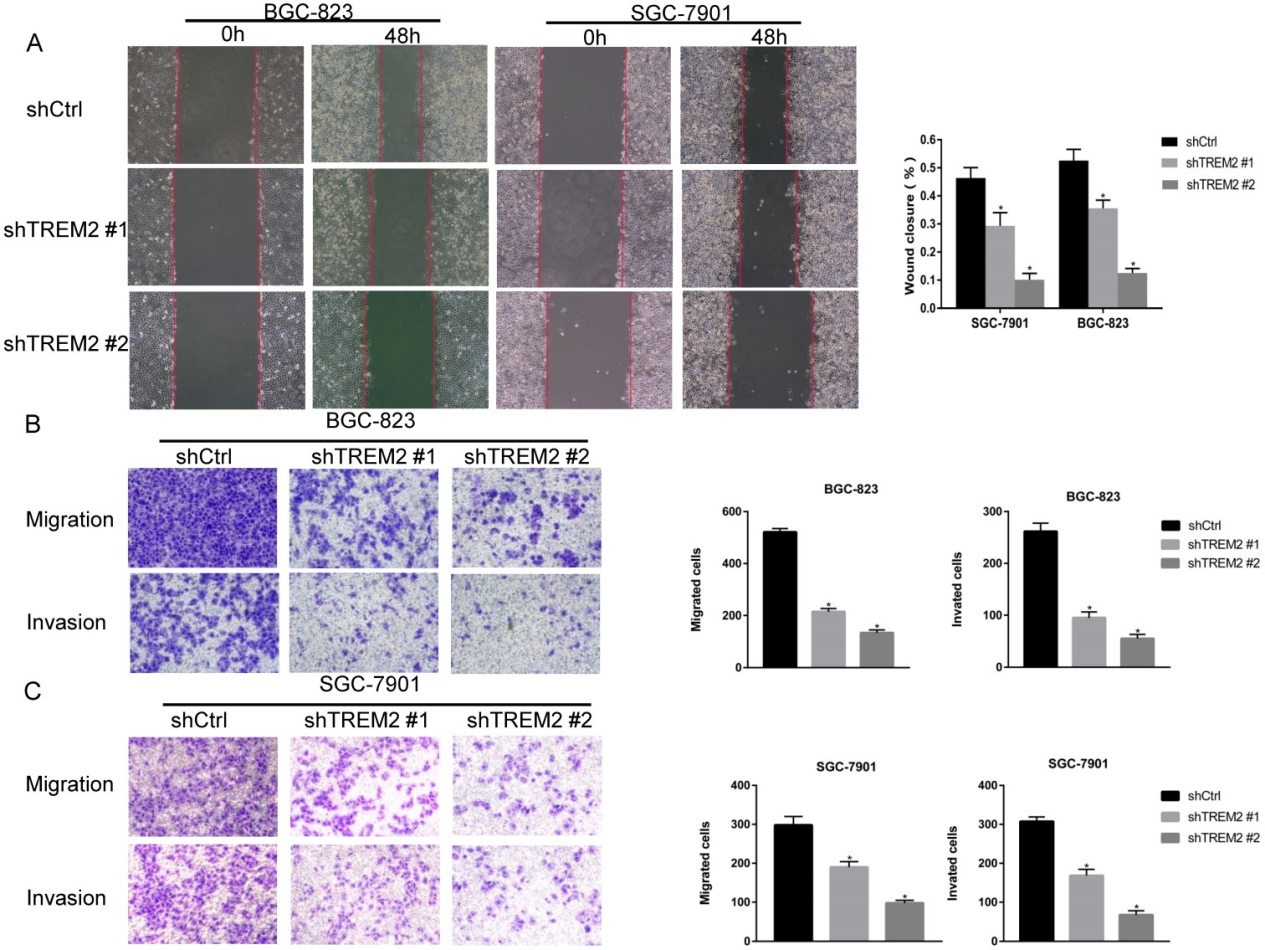
TREM2 affected the GC cell migration and invasion. Knockdown of TREM2, (A, B, C) the ability of migration assessed with wound healing assay and transwell migration assays (magnification, ×100), the ability of invasion measured by transwell invasion assays (magnification, X100). The statistical analysis was shown in the bar graphs (*, P<0.05, vs. control group; mean ± SD, n=3).

To investigate the role of TREM2 in GC, two kinds of shRNA against TREM2 were transfected into BGC-823 and SGC-7901 cell lines, the mRNA and protein expression levels of TREM2 were decreased by RT-PCR and Western blotting. The efficiency of shTREM2#2 knockdown TREM2 is higher than shTREM2#1 in BGC-823 and SGC-7901 cell lines (Figure 5C, 5D). Knockdown of TREM2 significantly inhibit cell proliferation ability compared to their corresponding control cell lines, by CCK-8, both in BGC-823 and SGC-7901 cell lines (Figure 5E). Wound healing assay and transwell migration assays were used to detect the effect of TREM2 on the migration of GC cells. The migration ability was inhibited when TREM2 silence (Figure 6A, 6B). The invasion ability also decreased after TREM2 konckdown by transwell invasion assays (Figure 6C). These results indicate that TREM2 is participates in the regulation of proliferation, migration, and invasion of GC.

### High expression of TREM2 promote EMT by PI3K/AKT pathway

**Figure 7:**
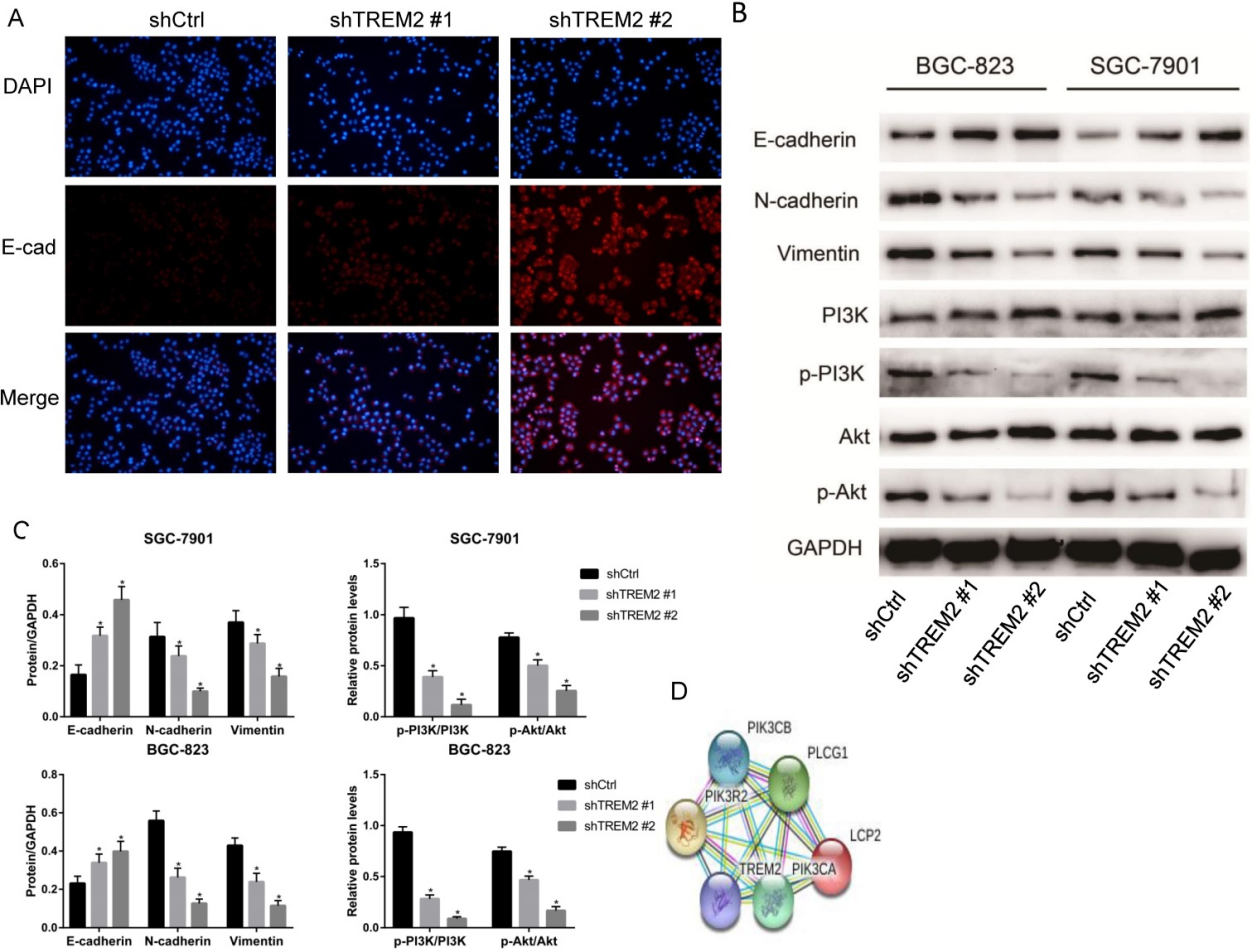
TREM2 promote EMT by PI3K/AKT pathway. (A) In GC cell line SGC-7901, the expression of E-cadherin was increased after knockdown of TREM2, measured by immunofluorescence staining (magnification, X100). (B) After knockdown of TREM2, the expression of E-cadherin was increased, the expression of N-cadherin, Vimentin, p-PI3K, p-AKT were decreased, the expression of PI3K and AKT not changed significantly. (C) The statistical analysis of proteins expression was shown in the bar graphs (*, P<0.05, vs. control group; mean ± SD, n=3). (D) Protein interaction network of TREM2 interacts with PIK3R2, PIK3CA, and PIK3CB proteins predicted by string website.

In the analysis of KEGG pathway enrichment of DEGs, EMT pathway accounted for the largest proportion in the GSE72305 and GSE103236 datasets, respectively. We therefore investigate whether key gene TREM2 is impact EMT process. The expression of E-cadherin increases when TREM2 expression decreased contrast of their corresponding control cell lines in SGC-7901 cell line observed by immunofluorescence (Figure 7A). Moreover, we used western blotting to detect the expression level of EMT-related protein in SGC-7901 and BGC-823 cell lines. After the konckdown of TREM2, the expression of E-cadherin elevated, and the expression of N-cadherin and Vimentin decreased (Figure 7B, 7C). The above data confirmed that TREM2 is participated in the EMT process of GC.

The string website predicted that TREM2 interacts with PIK3R2, PIK3CA, and PIK3CB proteins (Figure 7D), so we focused on the changes in the PI3K/AKT pathway. After knockdown of TREM2, there was no remarkable change in the expression of PI3K and AKT, but the expression of p-PI3K and p-AKT decreased in SGC-7901 and BGC-823 cell lines through western blotting (Figure 7B, 7C). As predicted, we found that PI3K/AKT pathway was inhibited when knockdown of TREM2. The PI3K/AKT pathway can regulate the EMT process, which has been proved in many kinds of tumors including GC. Combined with the above-mentioned outcomes, we believe that TREM2 can inhibit EMT through PI3K/AKT pathway.

### Knockdown of TREM2 inhibits GC cells metastasis *in vivo*

**Figure 8:**
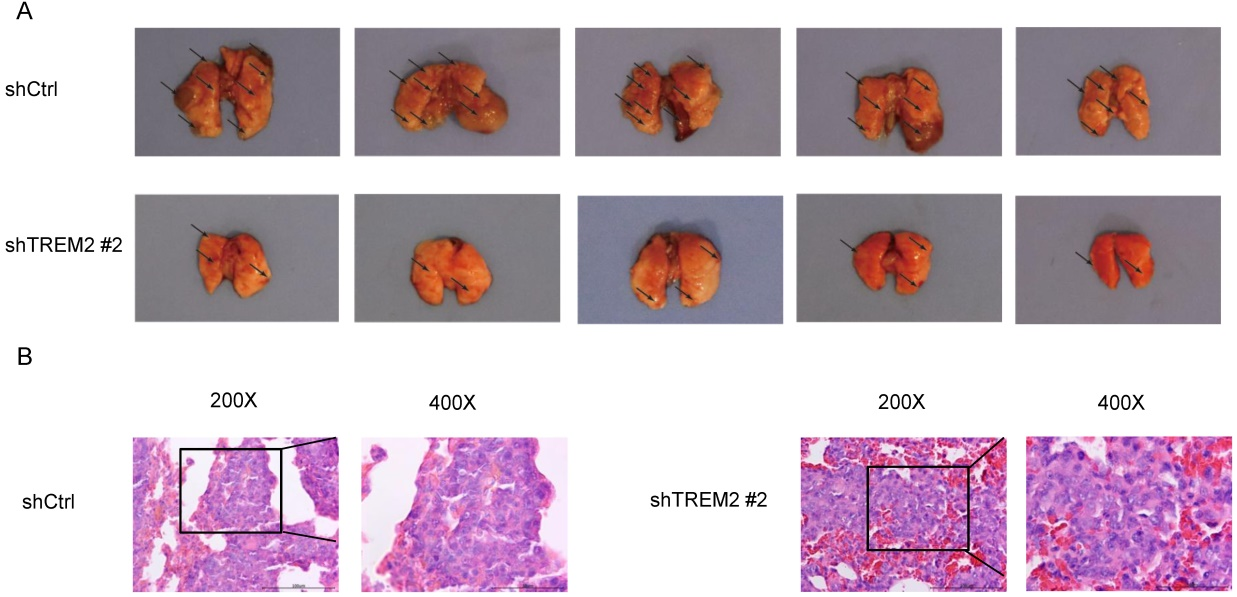
Knockdown of TREM2 inhibits GC cells metastasis *in vivo*. (A) The lung metastasis tumors about BGC-823 shCtrl group were increased than BGC-823 shTREM2# 2 group (n = 5/group). A representative photographs of gross lungs from indicated groups are shown (n = 5/group). (B) Representative H&E images of lung metastatic tumors.

To assess metastasis, BGC-823 shCtrl or BGC-823 shTREM2 #2 cells were injected into nude mice via the tail vein. The TREM2 high-expression group had more lung metastasis tumors than the TREM2 low-expression group. Representative photographs of obviously lungs and H&E images of lung metastatic tumors are shown for the indicated groups (Figure 8).

## Discussion

With the development of bioinformatics, we can obtain high-throughput data, such as mRNA profiles, DNA methylation status, non-coding RNA profiles, and SNPs, from GEO, TCGA, Oncomine public databases and determine the key genes and pathways involved in tumor development 3,7. This provides new targets for the molecular mechanisms of tumor research. Our research explored hub genes MMP9, KIF4A, and FBXO32 in the GSE72305 dataset and COL1A1, TIMP1, SPARC, COL5A2, SPP1, BGN, and COL4A1 in the GSE103236 dataset. Intersection genes TREM2, CTHRC1, BGN, NOX4, GPX3, HEYL, and SFRP4 from the GSE72305 and GSE103236 datasets, which were differentially expressed in the normal gastric mucosa than in gastric cancer cells and in lymph node free to lymph node metastatic GC. Therefore, these genes may play crucial roles in the development of GC.

To corroborate the outcomes of bioinformatics analysis, we reviewed the role of these genes in tumorigenesis and development, especially in GC. Previous analyses have reported these genes. All of the above genes are associated with the occurrence and development of GC in different degrees, which also proves the reliability of our analysis results. BGN, NOX4, SPARC, HEYL, SPP1, CTHRC1, SFRP4, FBXO32, KIF4A, MMP9, TIMP1 were related to the proliferation, invasion, prognosis, and recurrence of GC in different level and validated in many kind of tumors 25-37. The key gene GPX3 is downregulation in GC cell lines and samples because of abnormal promoter methylation, and related to lymph node metastasis 38. Cai et al. study proved that GPX3 affected migration and invasion through NFкB/Wnt5a/JNK pathway in GC 39. The key genes COL1A1, COL4A1, COL5A2 belong to the collagen family, which is a constituent of the ECM component of tumors, and is closely related to tumor proliferation, invasion, drug resistance, and prognosis 7,40-46. For example, the high expression of COL1A1 promotes cell proliferation, invasion, and migration in GC 42. Liu et al. research identified that COL1A1 can predict the prognosis of GC patients with *HP* (+) 43. COL5A2 has been identified as a hub gene in multiple biological analyses of GC, and enrich in EMT-related pathway 44. In another example, COL4A1 may confer trastuzumab resistance and promote gastric carcinoma recurrence in GC 45,46. Zhang et al. prior studies reported TREM2 is high expression in GC, and significantly associated with clinicopathological characteristics and prognosis 47. However, there are few studies on the specific mechanism of TREM2 affecting the occurrence and development of GC.

About 90% of gastric adenocarcinoma are associated with *HP* infection 48, TREM2 is not only associated with chronic inflammation and OS of GC patients, but also involve in carcinogenesis of some other tumors 11,17,19,47. Therefore, the synthesis of above features surprised us in GC. We carry out further cell studies to find the specific mechanisim about TREM2 involved in GC carcinogenesis, as extension and verification of bioinformatics analysis.

Our present study observed TREM2 is highly expressed in GC tissues and cell lines. In TCGA database, TREM2 is provides a key link of the clinicopathological features of GC (invasion depth, histologic grade, TNM stage, *HP* infection status, histologic grade and anatomic subdivision) in different data sets, which is consistent with previous research results. We were surprised to find that TREM2 is closely related to *HP* infection in GC. Moreover, after knockdown TREM2 expression in GC cell line BGC-823 and SGC-7901, the proliferation, migration, and invasion of GC cells were inhibited and lung metastasis was decreased in mice.

TREM2 is a cell surface receptor that does not have the ability to activate signals. Modern view, TREM2 binds to the ligand activating protein DNAX activating protein 12/Spleen tyrosine kinase (DAP12/Syk), then activate downstream signal pathways (PI3K, ERK, PLC pathway, etc.) to play a role 13. The prediction of string website suggests that TREM2 interacts with the proteins of PIK3R2, PIK3CA and PIK3CB. We assume TREM2/DAP12/Syk may play a further role through PI3K. On the other hand, EMT is correlation with initiation, invasion, metastasis of tumor based on different cellular states in cancer progression 49-51. The EMT-related pathway is the top altered pathway by DEGs KEGG enrichment analyses, suggesting the crucial role of EMT in GC initiation and progression. The activation of EMT depends on the stimulation of various extracellular signals (PI3K/AKT, Wnt, notch, TGF-β/Smad, ERK/MAPK, etc.) and the execution of EMT-TFs (snail, ZEB, and twist) 50,51. PI3K/AKT pathway promotes EMT process of cancer which has been confirmed in many experiments 50-54, such as mouse adult Schwann cells-derived CXCL5 can activated PI3K/AKT pathway and through EMT-TFs (Snail, Twist) to promote EMT in lung cancer 53. The lnc RNA PTTG3P can activate PI3K/AKT signaling by upregulating PTTG1, and affected cell cycle progression, cell apoptosis, and EMT-associated genes 54. We hypothesized TREM2/DAP12/Syk activated the PI3K / AKT pathway to promote the EMT process based on prediction of bioinformatics. As expected, our findings demonstrated this signaling pathway, and confirmed the prediction of bioinformatics, deepening the mechanism of inflammation related gene TREM2 in the pathogenesis of GC, and provided a new direction for the treatment of GC.

With the increasing appreciation of the relationship between inflammation of tumor, mounting evidence indicates that inflammation is a hallmark of tumors 48. The link between inflammation and GC has been confirmed in many experiments 55. For example, inflammation-related factors, consist of T lymphocytes, macrophages, cytokines, and pro-inflammatory chemokines, can promote the development of *HP* related GC 48, 56; Inflammatory fraction is associated with the prognosis of GC 57; In addition, amplification of inflammatory response can advance the occurrence of GC in the animal model 58. The above results are consistent with our idea inflammation related gene TREM2 can affects the malignant phenotype of gastric cancer. In previous analysis TREM2 is associated with *HP* infected, but exact mechanism about *HP* and TREM2 is unclear. When the gastric mucosa infected with *HP*, Syk can adjust the amplification of proinflammatory signaling 59. In another study, Syk associated with T1 tumors, lymphatic invasion, venous invasion and lymph node metastasis 60. TREM2 have functions after binds to DAP12/Syk activate downstream signal pathways. Whether *HP* infection can affect the expression of Syk through TREM2, and enlarge inflammation through TREM2, etc. needs further study.

In conclusion, we used a large database to find the novel gene in the development of GC and reviewed and analyzed the gene function. At the same time, the newly discovered inflammation related gene TREM2 has been verified to affect the EMT by PI3K/AKT pathway. This study extends the role of inflammation in the development of GC, facilitates us to better understand the relationship between inflammation and GC, and explores a possible therapeutic target for GC. However, the specific relationship and mechanism between *HP* and TREM2 need to be further explored.

## Supplementary Material

Supplementary figures and tables.

## Figures and Tables

**Table 1:** Functional role of 16 hub genes

Gene symbol	Full name	Function
BGN	biglycan	High expression of BGN enhance invasion and associated with a poor outcome in GC.
COL1A1	collagen, type I, alpha 1	High expression of COL1A1 promotes cell proliferation, migration, and invasion in GC.
COL4A1	collagen, type IV, alpha 1	COL4A1 may confer trastuzumab resistance and COL4A1 expression profiles can be reversed by drugs in GC.
COL5A2	collagen, type V, alpha 2	COL5A2 is a hub gene in most tumors, linked to angiogenesis, blood vessel development in MIBC.
NOX4	NADPH oxidase 4	NOX4 is involved in development and progression of GC, and as a new genetic target.
SPARC	secreted protein, acidic, cysteine-rich (osteonectin)	SPARC is associated with development and progression of GC, and predict GC recurrence.
HEYL	hes-related family bHLH transcription factor with YRPW motif-like	HEYL is target genes of Notch signaling pathway, involved in development and progression of most tumors.
SPP1	secreted phosphoprotein 1	High expression of SPP1 promoted proliferation, EMT process, but inhibited apoptosis of GC cells.
TIMP1	TIMP metallopeptidase inhibitor 1	High expression of TIMP1 is associated with proliferative potential and poor prognosis of GC.
CTHRC1	Collagen triple helix repeat containing 1	CTHRC1 is associated with invasion, metastasis and poor prognosis of GC.
TREM2	Triggering receptor expressed on myeloid cells 2	TREM2 have a role in chronic inflammations, related to poor prognosis of GC and progression of HCG, RCC, colon cancer.
SFRP4	Secreted frizzled-related protein 4	SFRP4 as modulators of Wnt signaling, and predicte chemotherapy response in resectable GC.
FBXO32	F-box protein 32	FBXO32 is a TGF-β/Smad signaling pathway target gene, can effected 5-FU resistance in GC.
GPX3	Glutathione peroxidase 3	GPX3 down-regulation in GC is due to aberrant promoter hypermethylation, suppressor of GC metastasis.
KIF4A	kinesin family member 4A	KIF4A is involved in development and progression of numerous types of cancer.
MMP9	Matrix metalloproteinase 9	High expression of MMP9 can promote metastasis in most tumors.

GC: gastric cancer; HCC: hepatocellular carcinoma; RCC: renal cell carcinoma; MIBC: muscle-invasive bladder cancer.

**Table 2:** Relationship between the expression of TREM2 and clinical characteristics in GC patients

Characteristic	No. tumors (%)			*P* value
	Total (n=393)	Low expression (n=192)	High expression (n=201)	
**Age (y)**				0.717
<60	118	56 (47.46)	62 (52.54)	
≥60	275	136 (49.45)	139 (50.55)	
**Sex**				0.764
Female	138	66 (47.83)	72 (52.17)	
Male	255	126 (49.41)	129 (50.59)	
**Pathologic T**				0.010*
Tis and T1	19	15 (78.95)	4 (21.05)	
T2	85	47 (55.29)	38 (44.71)	
T3	185	88 (47.57)	97 (52.43)	
T4	104	42 (40.38)	62 (59.62)	
**Pathologic N**				0.817
N0	125	60 (48.00)	65 (52.00)	
N1-3	268	132 (49.25)	136 (50.75)	
**Pathologic M**				0.435
M0	372	180 (48.39)	192 (51.61)	
M1	21	12 (57.14)	9 (42.86)	
**TNM stage**				0.014*
Stage I	53	35 (66.04)	18 (33.96)	
Stage II	135	58 (42.96)	77 (57.04)	
Stage III	172	79 (45.93)	93 (54.07)	
Stage IV	33	20 (60.61)	13 (39.39)	
**Histologic grade**				
G1	11	5 (45.45)	6 (54.55)	0.000*
G2	143	94 (65.73)	49 (34.27)	
G3	239	93 (38.91)	146 (61.09)	
**Histological type**				
Diffuse	71	27 (38.03)	44 (61.97)	0.015*
Intestinal	183	103 (56.28)	80 (43.72)	
NOS	139	62 (44.60)	77 (55.40)	
**Anatomic subdivision**				0.006*
Antrum	145	70 (48.28)	75 (51.72)	
Cardia	51	25 (49.02)	26 (50.98)	
Fundus	141	59 (41.84)	82 (58.16)	
GEJ	48	35 (72.92)	13 (27.08)	
NOS	8	3 (37.50)	5 (62.50)	

*p<0.05, statistically.

## Abbreviations

GC: gastric cancer
EMT: epithelial-mesenchymal transition
TCGA: The Cancer Genome Atlas
PPI: protein-protein interaction
GO: Gene Ontology
GEO: Gene Expression Omnibus
DEGs: differentially expressed genes
TREM2: Triggering receptor expressed on myeloid cells 2
KEGG: Kyoto Encyclopedia of Genes and Genomes
DAP12: DNAX activation protein 12
Syk: Spleen tyrosine kinase
BCA: Bicinchoninic acid
GEPIA: The Gene Expression Profiling Interactive Analysis
LMN: lymph node metastasis
OS: overall survival
FBS: fetal bovine serum
CCK8: Cell Counting Kit 8
RT-PCR: real-time polymerase chain reaction
SPSS: statistical package for the social sciences
SD: standard deviation
