# Supplementary material for: High expression of TREM2 promotes EMT via the PI3K/AKT pathway in gastric cancer: bioinformatics analysis and experimental verification
Source: J Cancer. 2021 Apr 2;12(11):3277–90. doi: 10.7150/jca.55077 (PMC8100818; doi:10.7150/jca.55077)
Supplement: Supplementary file 1 — Supplementary figures and tables. [file jcav12p3277s1.pdf]

High expression of TREM2 promotes EMT via the PI3K/AKT pathway in gastric cancer: bioinformatics analysis and experimental verification

Chunmei Li[1,2,3#], Xiaoming Hou[3#], Shuqiao Yuan[4#], Yigan Zhang[5], Wenzhen Yuan[6], Xiaoguang Liu[1,2,7], Juan Li[1,2,8], Yuping Wang[1,2], Quanlin Guan[6], Yongning Zhou[1,2*]

Figure S1 Expression of key genes in GC patients. The Gene Expression Profiling Interactive Analysis (GEPIA) was used to analyzed the expression of key genes, 15 of 16 genens overexpression in GC specimens compared to the normal specimens, except GPX3 is low expression.* means $p<0.05$, T means tumor tissues, N means normal tissues.

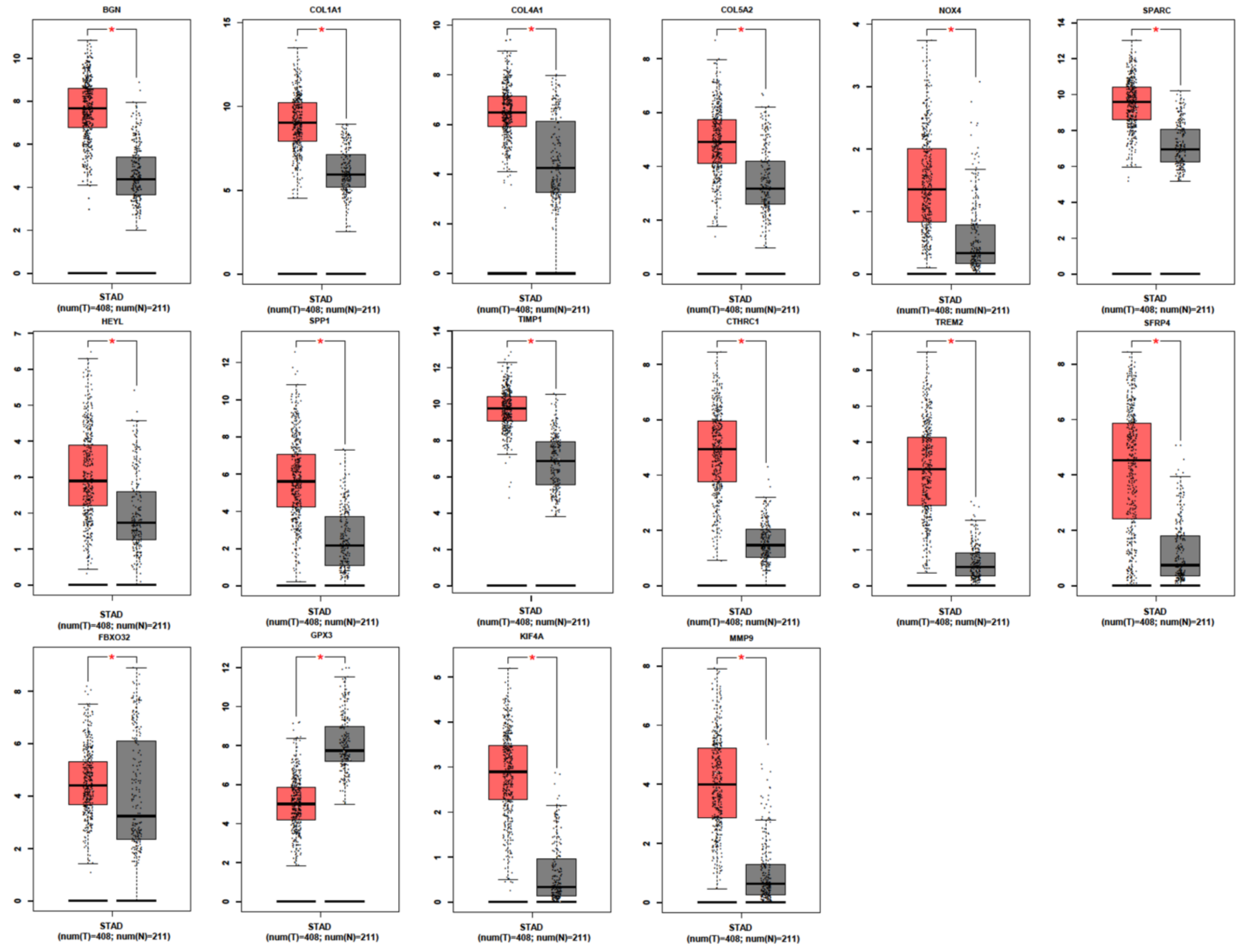

Table S1 Relationship between the expression of TREM2 and clinical characteristics in GC patients with *HP* state

Characteristic	No.tumors(%)			*P* value
	Total<br>n=175	Low expression<br>n=85	high expression<br>n=90	
Age（y）				0.924
<60	50	24(48.00)	26(52.00)	
≥60	125	61(48.80)	64(51.20)	
Sex				0.555
Female	51	23(45.10)	28(54.90)	
Male	124	62(50.00)	62(50.00)	
Pathologic T				0.038*
Tis and T1	10	9(90.00)	1(10.00)	
T2	41	20(48.78)	21(51.22)	
T3	79	33(41.77)	46(58.23)	
T4	45	23(51.11)	22(48.89)	
Pathologic N				0.217
N0	40	16(40.00)	24(60.00)	
N1-3	135	69(51.11)	66(48.89)	
Pathologic M				0.207
M0	168	80(47.62)	88(52.38)	
M1	7	5(71.43)	2(28.57)	
TNM stage				0.336
Stage I	24	15(62.50)	9(37.50)	
Stage II	48	21(43.75)	27(56.25)	
Stage III	86	39(45.35)	47(54.65)	
Stage IV	17	10(58.82)	7(41.18)	
Histologic grade				
G1	6	2(33.33)	4(66.67)	0.000*
G2	76	52(68.42)	24(31.58)	
G3	93	31(33.33)	62(66.67)	
Histological type				
Diffuse	33	10(30.30)	23(69.70)	0.063
Intestinal	118	63(53.39)	55(46.61)	
NOS	24	12(50.00)	12(50.00)	
Anatomic subdivision				0.431
Antrum	72	35(48.61)	37(51.39)	
Cardia	22	12(54.55)	10(45.45)	
Fundus	51	21(41.18)	30(58.82)	
GEJ	22	14(63.64)	8(36.36)	
NOS	8	3(37.50)	5(62.50)	
hp 状态				0.025*
hp +	20	5(25.00)	15(75.00)	
hp -	155	80(51.61)	75(48.39)	

*, $p<0.05$, statistically.
